# New Insights into the Phylogeny and Gene Context Analysis of Binder of Sperm Proteins (BSPs)

**DOI:** 10.1371/journal.pone.0137008

**Published:** 2015-09-02

**Authors:** Edith Serrano, Ana B. Martínez, Diana Arruga, Rosaura Pérez-Pé, Álvaro Sánchez-Ferrer, Teresa Muiño-Blanco, José A. Cebrián-Pérez

**Affiliations:** 1 Departamento de Bioquímica y Biología Molecular y Celular—Instituto Universitario de Investigación en Ciencias Ambientales de Aragón (IUCA), Facultad de Veterinaria, Universidad de Zaragoza, 50013, Zaragoza, Spain; 2 Department of Biochemistry and Molecular Biology-A, Faculty of Biology, Regional Campus of International Excellence “Campus Mare Nostrum”, University of Murcia, Campus Espinardo, E-30100, Murcia, Spain; 3 Murcia Biomedical Research Institute (IMIB-Arrixaca), 30120, Murcia, Spain; Cornell University, UNITED STATES

## Abstract

Seminal plasma (SP) proteins support the survival of spermatozoa acting not only at the plasma membrane but also by inhibition of capacitation, resulting in higher fertilizing ability. Among SP proteins, BSP (binder of sperm) proteins are the most studied, since they may be useful for the improvement of semen diluents, storage and subsequent fertilization results. However, an updated and detailed phylogenetic analysis of the BSP protein superfamily has not been carried out with all the sequences described in the main databases. The update view shows for the first time an equally distributed number of sequences between the three families: BSP, and their homologs 1 (BSPH1) and 2 (BSPH2). The BSP family is divided in four subfamilies, BSP1 subfamily being the predominant, followed by subfamilies BSP3, BSP5 and BSP2. BSPH proteins were found among placental mammals (Eutheria) belonging to the orders Proboscidea, Primates, Lagomorpha, Rodentia, Chiroptera, Perissodactyla and Cetartiodactyla. However, BSPH2 proteins were also found in the Scandentia order and Metatheria clade. This phylogenetic analysis, when combined with a gene context analysis, showed a completely new evolutionary scenario for the BSP superfamily of proteins with three defined different gene patterns, one for BSPs, one for BSPH1/BSPH2/ELSPBP1 and another one for BSPH1/BSPH2 without ELSPBP1. In addition, the study has permitted to define concise conserved blocks for each family (BSP, BSPH1 and BSPH2), which could be used for a more reliable assignment for the incoming sequences, for data curation of current databases, and for cloning new BSPs, as the one described in this paper, ram seminal vesicle 20 kDa protein (RSVP20, *Ovis aries* BSP5b).

## Introduction

Mammalian spermatozoa require extensive sperm plasma membrane remodelling during epididymal transit (epididymal maturation) and in the female reproductive tract (capacitation) to acquire their ability to fertilize [1,2]. Seminal plasma (SP) proteins have been recently shown to participate actively in both processes, not only in the survival of the spermatozoa but also inhibiting the capacitation. This combined effect results in higher fertilizing ability [3]. Among SP proteins, BSP (binder of sperm) proteins are the most studied, since they could represent up to 60% of total SP proteins in bovine [4,5]. The common characteristic of these BSP proteins is the presence of two fibronectin type II domains (FN2 domain), which confer them many binding properties, such as attachment to glycosaminoglycans [6–8], choline phospholipids [9], high and low-density lipoproteins [10,11] and gelatin [8,12]. Homologs of these proteins have been recently characterized in mouse and human [7,8], and named accordingly as mouse BSP homolog 1–3 (BSPH1-3) and human BSP homolog 1 (BSPH1).

Despite of their relevance in the capacitation process, only eighteen sequences have been previously compared to carry out their phylogenetic analysis [13,14]. These analyses showed that Fn2 domains found in BSP-related proteins have special features that distinguish them from non-BSP-related proteins and can be used to identify new BSP protein-related sequences. It has also been revealed that all BSP proteins can be grouped into three subfamilies: BSPH4, BSPH5 and BSPH6 whose names were later changed to BSP, BSPH1 and BSPH2 [14]. The objective of the present study was to present an updated comprehensive phylogenetic and gene context analysis in order to discover new putative BSP proteins, like ram seminal vesicle 20 kDa protein (RSVP20). These two analyses have shown a completely new evolutionary scenario for the BSP superfamily of proteins, different from that proposed earlier [14]. In addition, the study has permitted to define concise conserved blocks for each family (BSP, BSPH1 and BSPH2), which could be used for a more reliable assignment of the incoming sequences and data curation of current databases. Furthermore, the above *in silico* studies have been validated by cloning and expression the gene corresponding to *Ovis aries* RSVP20, since no previous BSP5 protein has been cloned.

## Results

### Phylogenetic analysis

**Fig 1:**
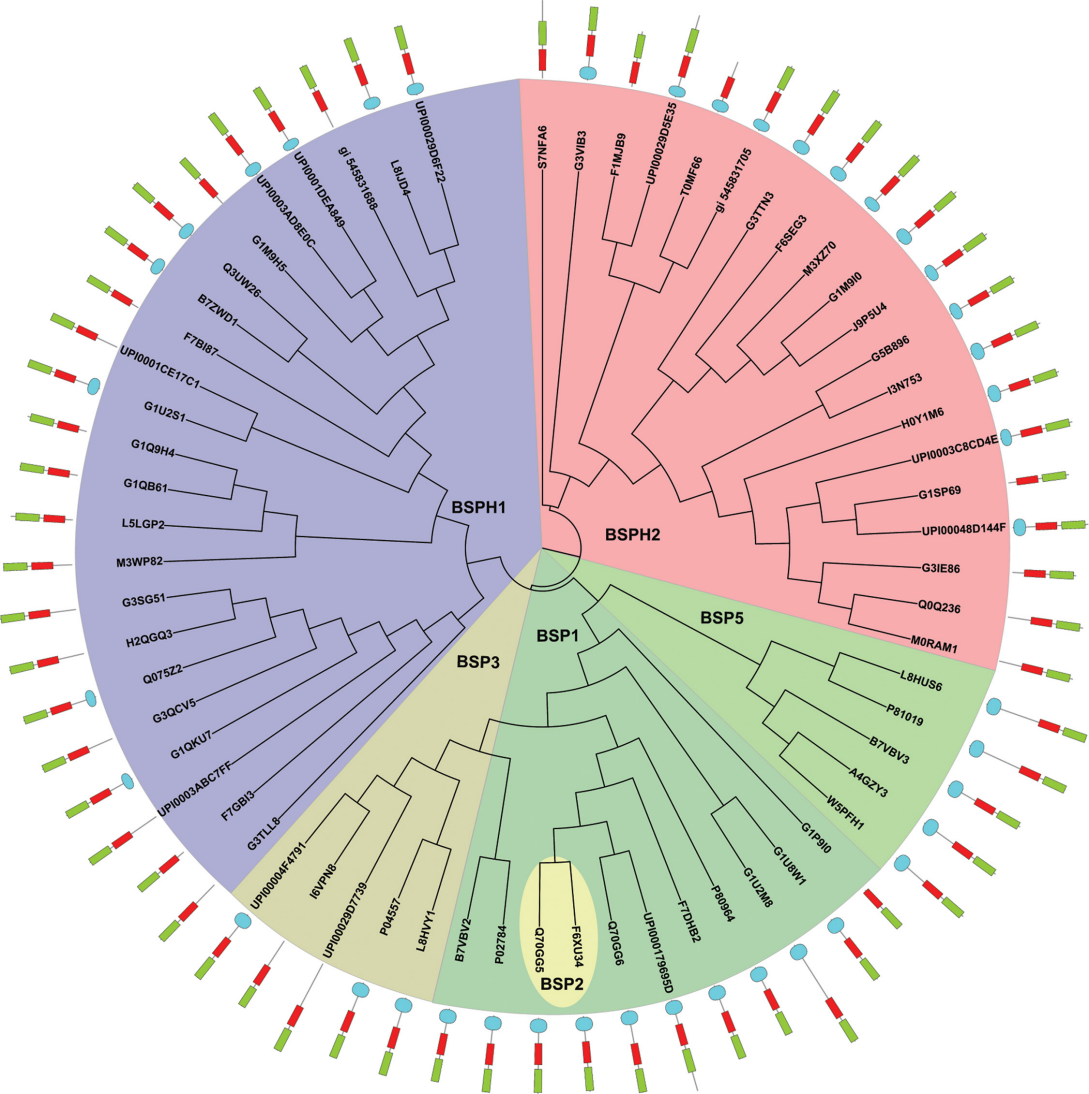
Phylogenetic analysis of Binder of Sperm Proteins. BSP proteins are divided into the main subfamilies: BSP (green), BSPH1 (purple) and BSPH2 (hot pink). In addition, the BSP subfamily is composed of four different clades corresponding to BSP1 (olive green), BSP2 (yellow green), BSP3 (camouflage green) and BSP5 (light green). The structures behind each protein code represent domain composition: signal peptide (cyan), 1FN2 (red) and 2FN2 (fluorescent green). C-terminal section of the protein is the outer part of the domain representation. The neighbor-joining (NJ) tree was obtained from 1000 replicates. Bootstrap values are indicated in S1 Fig.

The phylogenetic study was carried out using the unique 64 sequences found in the UniProt, Ensembl and NCBI databases. The tree obtained (Fig 1 and S1 Fig) shows an equally distributed number of sequences between the 3 families (BSP, BSPH1 and BSPH2). However, in BSP family, the predominant is BSP1 subfamily, followed by subfamilies BSP3 and BSP5. Of note are the only two equine sequences (Q70GG5 and F6XU34) found in the BSP2 subfamily (Fig 1, yellow green), which has evolved in parallel to the other two BSP1 sequences described in *Equus caballus* (Table 1, Fig 1). The BSP1 group (Fig 1, olive green) is basically formed by BSP proteins from leporidae (UniProt codes: G1U8W1 and G1U2M8), equidae (UniProt code: Q70GG5), suidae (UniProt code: P80964) and bovidae (UniProt codes: P02784 and B7VBV2), whereas the BSP3 group (Fig 1, camouflage green) is restricted to 4 bovinae sequences and a new *O*. *aries* BSP (UniProt code: UPI00029D7739). BSP5 clade (Fig 1, light green) is formed only by sequences of the bovinae and caprinae subfamilies, in which ram RSVP20 and RSVP22 [15] are located close to *Bos taurus* BSP5 (UniProt code: P81019), a new *B*. *taurus* BSP5 (UniProt code: L8HUS6) and also a new *Ovis aries* uncharacterized protein (UniProt code: W5PFH1), giving rise to a new defined clade, compared with previously described trees [13,14].

**Table 1:** Updated nomenclature for Binder of Sperm Proteins.

UniProt or NCBI accession numbers	Species	Existing gene symbol UniProt	Proposed gene symbol
P02784 (SFP1_BOVIN) ^§^	*Bos taurus* (Bt)	BSP1	BSP1
L8HVY1	*Bos mutus*	M91_09268	BSP3
L8HUS6	*Bos mutus*	M91_09267	BSP5
P04557 (SFP3_BOVIN)	*Bos taurus*	BSP3	BSP3a
UPI00004F4791	*Bos taurus*		BSP3b
P81019 (SFP4_BOVIN)	*Bos taurus*	BSP5	BSP5
I6VPN8	*Bubalus bubalis*	SPA3	BSP3
F7DHB2	*Equus caballus* (Ec)	LOC100629397	BSP1
Q70GG6 (P81121,Q71U23)	*Equus caballus*	sp1/BSP1	BSP1
UPI000179695D	*Equus caballus*		BSP1
Q70GG5 (F6XU34,Q70GG4)	*Equus caballus*	spneu	BSP2a
F6XU34	*Equus caballus*	BSP2	BSP2b
G1P9I0	*Myotis lucifugus*		BSP1
G1U8W1	*Oryctolagus cuniculus* (Oc)		BSP1a
G1U2M8 (O97690)	*Oryctolagus cuniculus*	BSP1	BSP1b
B7VBV2	*Ovis aries* (Oa)	RSVP14/BSP1	BSP1
UPI00029D7739	*Ovis aries*		BSP3
W5PFH1	*Ovis aries*	BSP5	BSP5a
A4GZY3	*Ovis aries*	RSVP20	BSP5b
B7VBV3 (W5QJB1)	*Ovis aries*	RSVP22	BSP5c
P80964 (PB1_PIG)	*Sus scrofa* (Ss)	BSP1	BSP1
G1M9H5 (D2H089)	*Aliuropoda melanoleuca* (Am)	BSPH1	BSPH1a
UPI0001DEA849	*Aliuropoda melanoleuca*		BSPH1b
L8IJD4	*Bos mutus*	M91_16052	BSPH1
F7GBI3	*Callithrix jacchus*	BSPH1	BSPH1
UPI0003AD8E0C (F1PR45)	*Canis familiaris* (Cf)		BSPH1
F7BI87	*Equus caballus*	BSPH1	BSPH1
M3WP82	*Felis catus*	BSPH1	BSPH1
G3SG51	*Gorilla gorilla*	101138349	BSPH1a
G3QCV5	*Gorilla gorilla*	101138349	BSPH1b
Q075Z2	*Homo sapiens* (Hs)	BSPH1	BSPH1
G3TLL8	*Loxodonta africana* (La)	BSPH1	BSPH1
UPI0003ABC7FF (G7PY10,F7EM06)	*Macaca fascicularis*		BSPH1
B7ZWD1	*Mus musculus* (Mm)	BSPH1	BSPH1a
Q3UW26	*Mus musculus*	BSPH1	BSPH1b
L5LGP2	*Myotis davidii*	MDA_GLEAN10005915	BSPH1
G1QB61	*Myotis lucifugus*		BSPH1
G1Q9H4	*Myotis lucifugus*		BSPH1
G1QKU7	*Nomascus leucogenys*	BSPH1	BSPH1
G1U2S1	*Oryctolagus cuniculus*	BSPH1	BSPH1a
UPI0001CE17C1	*Oryctolagus cuniculus*		BSPH1b
UPI00029D6F22 (W5PPG8)	*Ovis aries*		BSPH1
H2QGQ3	*Pan troglodites*	BSPH1	BSPH1
gi_545831688	*Sus scrofa*	BSPH1	BSPH1
F1MJB9 (L8IJZ5)	*Bos taurus*	BSPH2	BSPH2
T0MF66	*Camelus ferus*	CB1_000516002	BSPH2
J9P5U4	*Canis familiaris*		BSPH2
G3IE86	*Cricetulus griseus*	I79_022030	BSPH2
F6SEG3	*Equus caballus*		BSPH2
G5B896	*Heterocephalus glaber*	GW7_05342	BSPH2
G3TTN3	*Loxodonta africana*		BSPH2
Q0Q236	*Mus musculus*	BSPH2	BSPH2
M3XZ70	*Mustela putorius furo (Mpf)*	BSPH1	BSPH2
S7NFA6	*Myotis brandtii*	D623_10017573	BSPH2
G1SP69	*Oryctolagus cuniculus*	LOC100341751	BSPH2
UPI00048D144F	*Oryctolagus cuniculus*		BSPH2
H0Y1M6	*Otolemur garnettii*	BSPH1	BSPH2
UPI00029D5E35 (W5PPC7)	*Ovis aries*		BSPH2
M0RAM1	*Rattus novergicus (Rn)*	BSPH2	BSPH2
G3VIB3	*Sarcophilus harrisii (Sh)*	BSPH1	BSPH2
I3N753	*Spermophilus tridecemlineatus*	BSPH1	BSPH2
gi_545831705	*Sus scrofa*	BSP-30 kDa like	BSPH2
UPI0003C8CD4E	*Tupaia chinensis*		BSPH2

^§^Accession numbers in parenthesis mean duplication of the same protein in UniProt

Updated information about BSPH1 and BSPH2 subfamilies is also provided in Fig 1. Both types of BSPH proteins were found among placental mammals (Eutheria) belonging to the orders Proboscidea, Primates, Lagomorpha, Rodentia, Chiroptera, Perissodactyla, Artiodactyla and Cetacea. However, BSPH2 proteins were also found in the Chinese tree shrew (*Tupaia chinensis*, UniProt code: UPI0003C8CD4E) belonging to the Scandentia Order and in the metatherian Tasmanian devil (UniProt code: G3VIB3), belonging to the Scandentia and Dasyuromorphia orders, respectively. It is also noteworthy that two new sequences corresponding to *O*. *aries* BSPH1 (Uniprot code: UPI00029D6F22) and BSPH2 (UniProt code: UPI00029D5E35) were found close to their corresponding bovinae homologues (Uniprot codes: L8IJD4 and F1MJB9, respectively).

### Analysis of conserved sequence blocks

**Fig 2:**
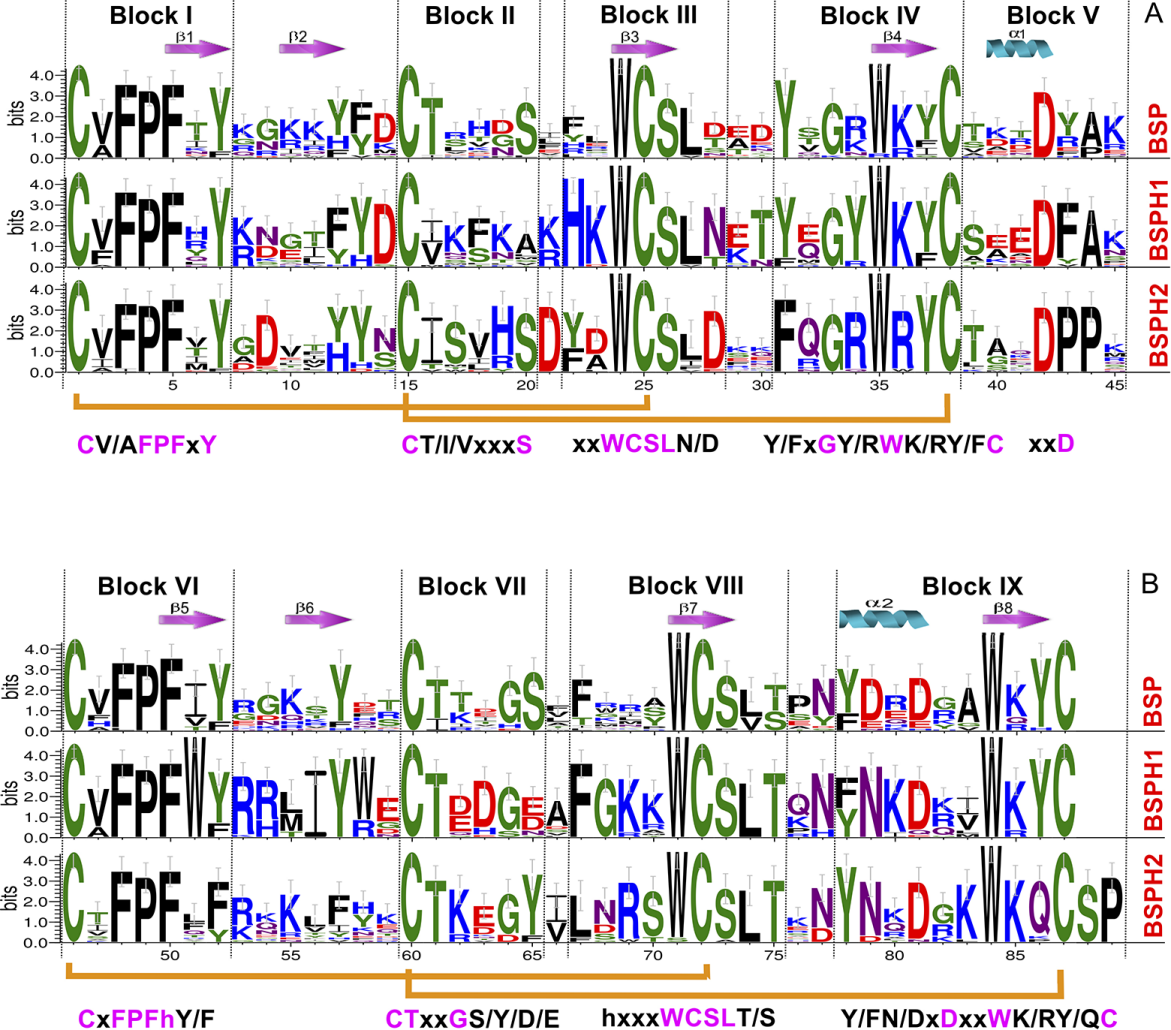
Conserved sequence blocks of two FN2 domains in Binder of Sperm Proteins. (A) Logo representation of 1FN2 domain (N-ter domain). (B) Logo representation of 2FN2 domain (C-ter domain). Symbols above sequences represent the secondary structure; springs represent helices and arrows represent β-strands. Orange lines connecting conserved cysteines correspond to disulphide bonds. Consensus sequences are shown below each conserved block. h stands for hydrophobic amino acid.

In order to fully understand the results described in Fig 1, a detailed study of the conserved sequence blocks was carried out using WebLogo3 [16] and ESPript [17] representations of the three different subfamilies (BSP, BSPH1 and BSPH2), and illustrated in base of RSVP20 sequence, corresponding to a non characterized BSP5 protein (S2 Fig). Nine conserved blocks were found (Fig 2), corresponding to two tandem FN2 domains with four blocks each and a linker block in between. Surprisingly, the C-ter FN2 (2FN2) domain is four amino acids longer than N-ter FN2 (1FN2) (Fig 2).

**Fig 3:**
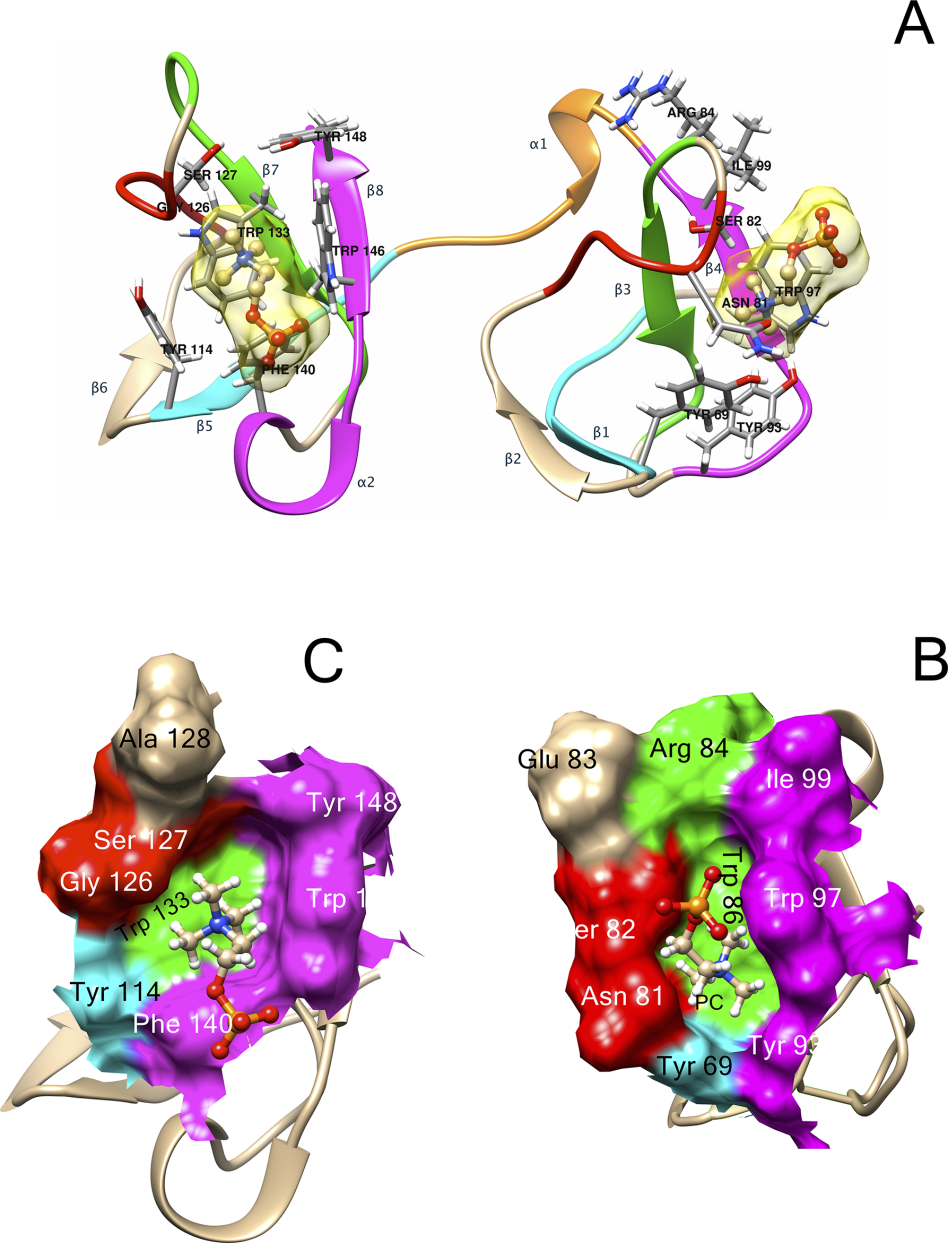
*In silico* analysis of Binder of Sperm Proteins. (A) Ribbon representation of the modelled *Ovis aries* RSVP20 protein. Different colors represent different conserved blocks: Cyan (Block I and VI), red (Block II and VII), green (Block III and VIII), magenta (block IV and IX) and orange (Block V). Amino acids forming both phosphorylcholine (PC) binding sites are shown in sticks and labeled. A PC molecule in the proposed binding site is shown in ball and stick representation. (B) Surface representation of 1FN2 PC binding site. (C) Surface representation of 2FN2 PC binding site. Amino acids involved in both binding sites are labeled according RSPV20 numbering and colours are the same as blocks described in (A) (see also S2 Fig).

The first block of the 1FN2 domain (Block I) has a consensus sequence **C** V/A **FPF**x**Y** (Fig 2A). This block includes the first conserved cysteine (C1 in Fig 2A, C63 in RSVP20, S2 Fig) involved in one of the two conserved disulfide bonds (C1-C25, Fig 2A), and the first β strand (F5-Y7 in Fig 2A, F67-Y69 in RSVP20, S2 Fig) of the double stranded antiparallel β sheet (β1-β2) (Fig 3A, right cyan and tan β-strands, respectively). Curiously, β2 (positions 10–12 in Fig 2A, R72-Y74 in RSVP20, S2 Fig; Fig 3A right, tan β-strand) does not define a clear conserved block either in BSPs or in BSPHs (Fig 2A). Block II (**C** T/I/V xxx**S**) is located the intervening β2-β3 loop (positions 15–20 in Fig 2A, C77-S82 in RSVP20, S2 Fig: Fig 3A right, red loop) and its last two amino acids form (positions 19–20 in Fig 2A, N81-S82 in RSVP20) together with the Block I conserved Y (Y7 in Fig 2A, Y69 in RSVP20, S2 Fig), one of the walls of the phosphorylcholine (PC) binding pocket 1 (Fig 3B, red and cyan, respectively). The end of this block usually shows a conserved serine (S82 in RSVP20), except for the BSPH1 clade where it is replaced basically by another small amino acid (i.e., alanine). This block II also contains the second conserved cysteine (C15 in Fig 2, C77 in RSVP20, S2 Fig). The strictly conserved tryptophan (W24 in Fig 2A, W86 in RSVP20, S2 Fig) responsible for the cation-*π* interaction between the quaternary ammonium group of PC and its indole ring (Figs 2A and 3A right and 3B, green) is found in Block III (xx**WCSL** N/D), just at the beginning of β3 (**WCS**), where the third conserved cysteine (C25 in Fig 2, C87 in RSVP20, S2 Fig) is placed. The latter tryptophan (W86 in RSVP20) lines the bottom of the PC binding pocket 1 (Fig 3B, green), whereas the top position is covered by a non-conserved amino acid located in the same Block III but two positions before (R84 in RSVP20) (Figs 2A and 3A right and 3B, green). However, this top position is a strictly conserved histidine in BSPH1 (position H22, Fig 2A), giving rise together with the rest of block III to a clear fingerprint for BSPH1s (**HKWCSLN**) (Fig 2A). The last block of the 1FN2 domain (Block IV, Y/F x**G** Y/R **W** K/R Y/F **C**) forms the opposite wall to block II in the PC binding pocket 1 (Y93, W97 and I99 in RSVP20, respectively) (Fig 3B, magenta), the conserved β4 (**W** K/R Y/F) (Fig 2A) and the last conserved cysteine 4 (C38 Fig 2, C100 in RSVP20; S2 Fig). Molecular docking analysis of RSVP20 with PC showed a binding energy to this 1FN2 domain binding pocket of -5.99 kcal/mol, which is in the range of previously described values of murine BSPH1 and BSPH2 (both with a value of -3.8 kcal/mol) [8]. The interdomain or linker segment (Block V, Fig 3A, orange) shows only a clear conserved aspartic at the end of α1 (xx**D**, D42 in Fig 2A, D104 in RSVP20, S2 Fig). However, this domain displays a clear fingerprint for BSPH2s (**DPP**) together with Block IV (**F**x**GRWRYC**). This Block V has been described as being involved in the dimerization process together with edges of the β2-β3 loop [18].

**Fig 4:**
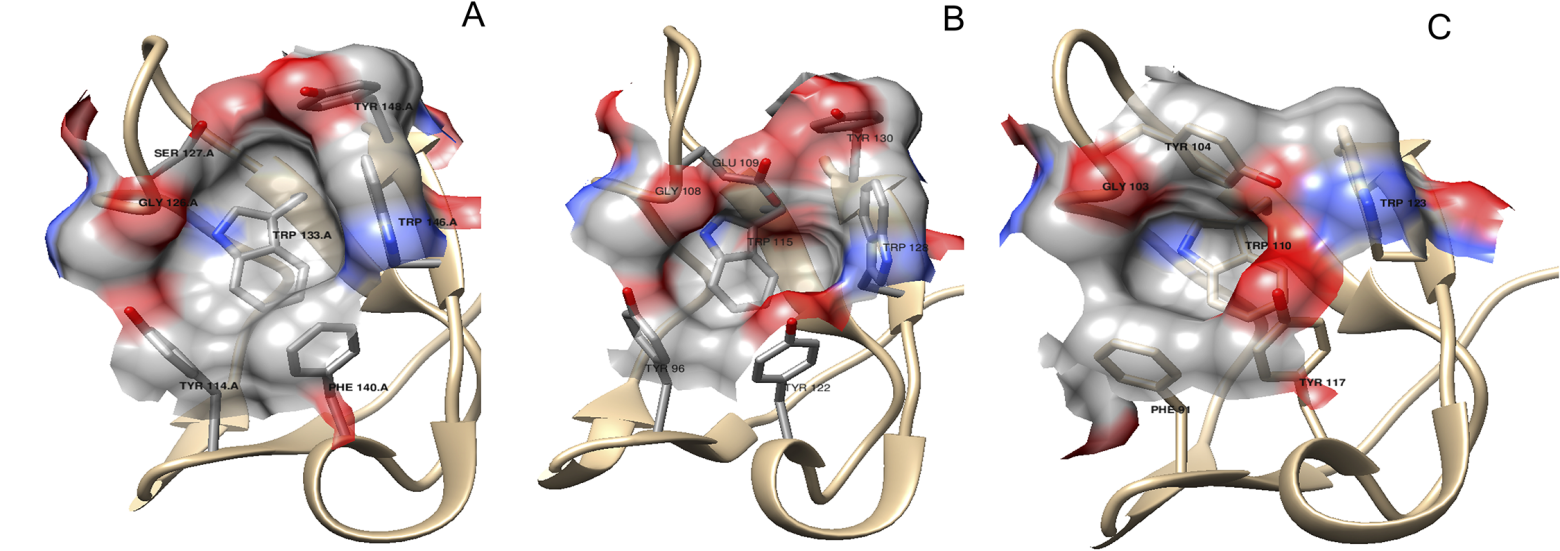
*In silico* analysis of 2FN2 phosphorylcholine binding site. The size of PC binding site 2 is reduced from (A) RSVP20 (BSP5b) to (B) murine BSPH1 and from the latter to (C) murine BSPH2. The first decrease in size is due to a change from a serine (S127 in RSVP20) to a glutamic acid (E109 in mBSPH1). The second decrease in size is due to the change of the latter E109 to a tyrosine (Y104 in mBSPH2). Cartoon representation of the backbone is in tan and surfaces are displayed in CPK (carbon in grey, oxygen in red and nitrogen in blue).

Block VI (**C**x**FPF**h Y/F, where h stands for hydrophobic amino acid) at the 2FN2 domain is similar to that of Block I (Fig 3A left, cyan). However, this latter block is clearly another fingerprint in BSPH1 proteins (**C**h**FPFWY**), where a strictly conserved tryptophan in the middle of β5 appeared, giving rise to a clear hydrophobic tract (V/A **FPFW**) (Fig 2B). The last hydrophobic amino acid of Block VI (position 52 in Fig 2B, and Y114 in RSVP20; S2 Fig; Fig 3C, cyan) and the last two amino acids of Block VII (GTxxG S/Y/D/E) (positions 64–65 in Fig 2B, G126 and S127 in RSVP20, S2 Fig; Fig 3C, red) form one of the walls of the PC binding pocket 2. The bottom of this pocket 2 is occupied by the completely conserved tryptophan (W71 in Fig 2B, W133 in RSVP20, S2 Fig; Fig 3C, green) of Block VIII (hxxx**WCSL** T/S). This latter block is also another fingerprint for the BSPH1 family (**FGKKWCSLT**). Two differences are clearly shown when this block VIII is compared with the homolog block (Block III) in the 1FN2 domain. The first is related with the size, as Block VIII is two amino acids longer than Block III (Fig 2). The second is related with the fact that only one amino acid (W71 in Fig 2B, W133 in RSVP20, S2 Fig) of this Block VIII is involved in the PC binding pocket 2 structure (Fig 3C, green), whereas two amino acids (position 22 and W24 in Fig 2A, R84 and W86 in RSVP20, S2 Fig) of Block III are associated with the PC binding site 1 structure (Fig 3B, green). Finally, Block IX (Y/F N/D x**D**xx**W** K/R Y/Q **C**) at C-ter delimits the opposite wall to Block VI and VII at the PC binding pocket 2 (Fig 3C, magenta). In fact, the wall is formed by the first amino acid of α2 (Y/F N/D x **D**; position 78 in Fig 2B, F140 in RSVP20, S2 Fig), and the first and the last amino acid of β8 (**W** K/R Y/Q; W84 and position 86 in Fig 2B, W146 and Y148 in RSVP20, S2 Fig, respectively) (Fig 3C, magenta). This Block IX also differs in size from the homolog in 1FN2, this block again being two amino acids longer (Fig 2). It is noteworthy that this Block IX is an unquestionable BSPH2 fingerprint (**YN**x**D**x**KWK**QC), which has to be supplemented with two strictly conserved amino acids (serine and proline, **SP**) at the end of the 2FN2 motif (Fig 2B). This conserved C-ter extension after 2FN2 is shown neither in BSPs nor BSPH1s (Fig 2B). The 2FN2 pocket appeared to bind PC with an affinity of -5.29 kcal/mol, which is in the range of murine BSPH1 (-3.5 kcal/mol) but higher than that described for murine BSPH2 (-2.8 kcal/mol) [8]. These docking results could be explained by the decreasing size of the PC binding pocket 2 from BSPs (i.e., RSVP20) to mBSPH1 and from mBSPH1 to mBSPH2 (Fig 4, S2 Fig triangle). The first decrease in size is due to a change from a serine (S127 in RSVP20) to a glutamic acid (E109 in mBSPH1) (Fig 4A and 4B, S2 Fig triangle). The second is due to the change of the latter glutamic acid (E109 in mBSPH1) for a tyrosine (Y104 in mBSPH2), whose electronic density connects with that of Y117, closing the binding site (Fig 4B and 4C, S2 Fig triangle).

### Gene context analysis

**Fig 5:**
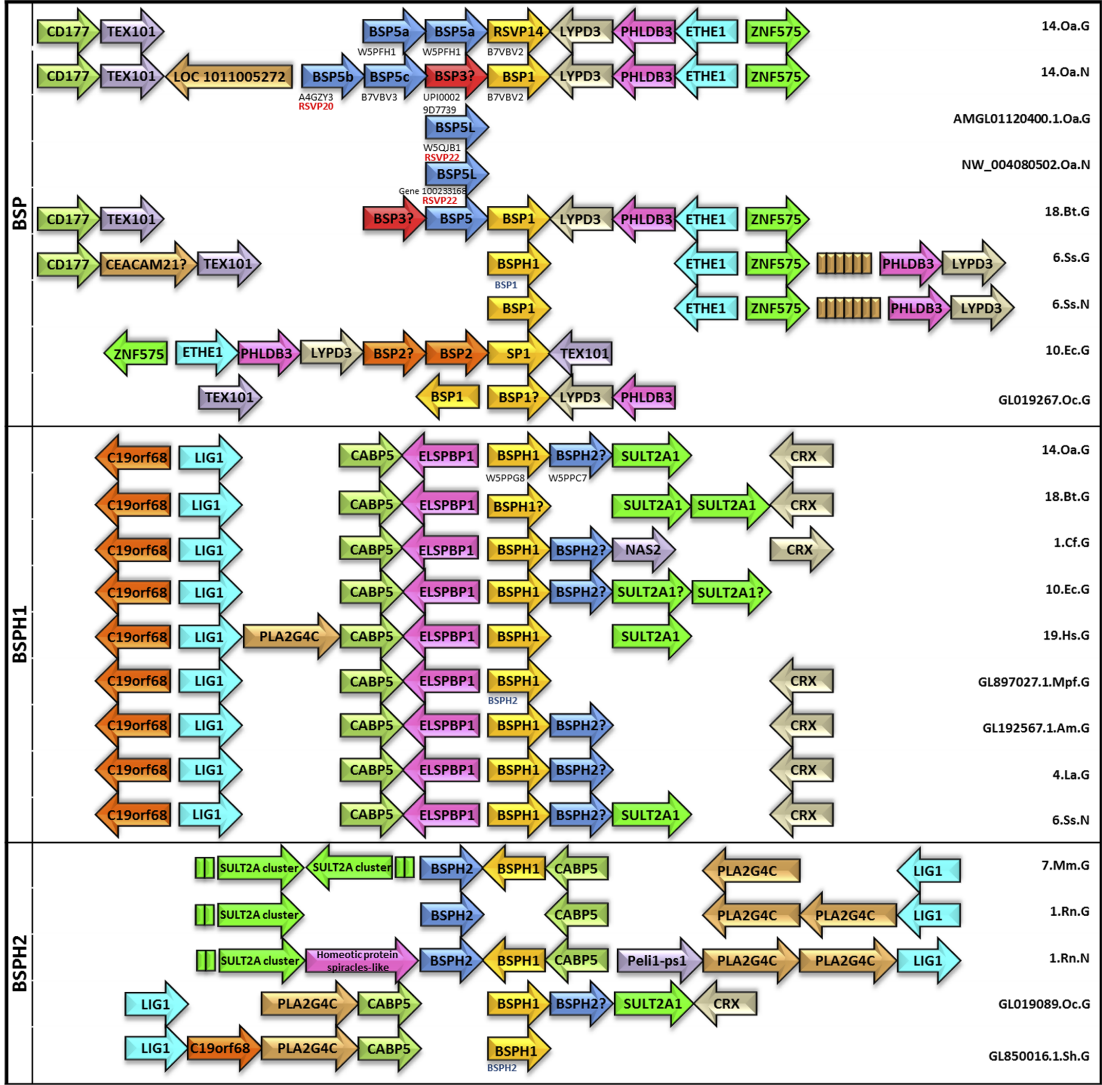
Genome context analysis for Binder of Sperm Proteins. The three different patterns (BSP, BSPH1 and BSPH2)were found in Genomicus (G) or in NCBI (N) databases. Abbreviations on the right hand of each box represent chromosome/scaffold number (i.e. 14), species (i.e. Ov for *Ovis aries*) and database (i.e. G, Genomicus), respectively. In *Ovis aries*, letters bellow arrows mean the corresponding UniProt Code and letters in red correspond to trivial name. Letters in blue bellow arrows represent the correct name of missanotated proteins. Names with? at the end mean new BSP proteins not previously described. See text for gene and Table 1 for species abbreviations, respectively.

To better understand the role of the BSP family, a gene context analysis was carried out to determine its potential operonic associations, using the Genomicus and NCBI databases (Fig 5). The BSP subfamily, to which RSVP20 belongs, shows a regular pattern in which BSP proteins are flanked on one side by the gene cluster formed by CD177 (GPI-linked surface protein) and TEX101 (Testis EXpressed 101 protein, found in epididymal sperm plasma membrane and which may play an important role in sperm-egg interaction) on one side, and by LYPD3 (LY6/Plaur Domain containing protein 3), PHLDB3 (Pleckstrin Homology-Like Domain, family B, member 3), ETHE1 (ETHylmalonic Encephalopathy 1, a mitochondrial matrix sulfur dioxygenase) and ZNF575 (ZiNc Finger protein 575, involved in transcriptional regulation) on the other side. Curiously, in *O*. *aries* BSP gene distribution is different in Genomicus and NCBI databases (Fig 5) and leads to the discovery of two new sequences in addition to the known RSVP14 (UniProt code: B7VBV2), RSVP20 (UniProt code: A4GZY3) and RSVP22 (UniProt code: B7VBV3). Thus, in Genomicus (Fig 5, BSP box, 14.Oa.G), a new BSP5 sequence (BSP5a UniProt code: W5PFH1) is found twice, and followed by the known RSVP14 gene in chromosome 14 without the presence of RSVP20 and RSVP22 sequences, respectively. However, in NCBI (Fig 5, BSP box, 14.Oa.N), four BSP genes are found, corresponding to BSP5 (RSVP20, BSP5b), LOC101107624 (seminal plasma protein BSP-30 KDa like = RSVP22 = B7VBV3, BSP5c), LOC101105521 (seminal plasma protein A3-like, which is UniProt UPI00029D7739) and BSP1 (RSVP14), respectively. Phylogenetic analysis of UPI00029D7739 gave rise, for the first time, to a possible existence of a BSP3 protein in *O*. *aries*, as shown in Fig 1. In addition, both the Genomicus (Fig 5, BSP box, AMGL01120400.1.Oa.G) and NCBI (Fig 5, BPS box, NW_004080502.Oa.N) databases show the existence of a BSP5L gene in AMGL01120400.1 and NW_004080502 scaffolds, respectively. Blast and phylogenetic analysis (Fig 1) showed that the BSP5L gene product (UniProt W5QJB1) is in fact RSVP22 protein.

These discrepancies between databases for BSP pattern are also shown in *Sus scrofa* chromosome 6. Genomicus names UniProt code P80964 as BSPH1 (Fig 5, BSP box, 6.Ss.G), whereas NCBI names it BSP1 (the correct name) (Fig 5, BSP box, 6.Ss.N). In addition, the number of genes in the CD177-TEX101 side and the ETHE1-LYPD3 side are different in chromosome 6 (Fig 5, BSP box, 6.Ss.G/N). Furthermore, this gene context analysis also shows the presence of two BSP1 genes in rabbit GL019267 scaffold (Fig 5, BSP box, GL019267) (UniProt codes: G1U2M8 and G1U8W1, respectively). No discrepancies from known information were found in *Bos taurus* (Fig 5, BSP box, 18.Bt.G), which has BSP1 (UniProt code: P02784), BSP3 (UniProt code: P04557) and BSP5 (UniProt code: P81019). An updated nomenclature for the BSP gene family in the different databases is proposed in Table 1.

A second gene context group was found to be associated with BSPH1 genes (Fig 5, BSPH1 box). In this group, BSPH1 is flanked on one side by C19orf68, LIG1 (ATP-dependent DNA LIGase I), CABP5 (Calcium Binding Protein 5) and ELSPBP1 (EpididymaL Sperm Binding Protein 1), and on the other side by BSPH2, SULT2A1 (SULfoTransferase 2A1) and CRX (Cone-Rod homeoboX) (Fig 5, BSPH1 box). This pattern is also associated with *O*. *aries* BSPHs, with BSPH1 (UniProt code: W5PPG8) and BSPH2 (UniProt code: W5PPC7) being described for the first time in chromosome 14 (Fig 5, BSPH1 box, 14.Oa.G). New BSPH2s were also found in this gene context in dog (UniProt code: J9P5V4), horse (UniProt code: F6SEG3), giant panda (UniProt code: G1M910), African bush elephant (UniProt code: G3TTN3) and pig (NCBI code: gi_545831705) (Fig 5, BSPH1 box, 1.Cf.G, 10.Ec.G, GL192567.1.Am.G, 4.La.G and 6.Ss.N, respectively). Interestingly, a misannotation was found in ferret (*Mustela putorius furo*) GL897027.1 scaffold (Fig 5, BSPH1 box, GL897027.1.Mpf.G), where M3XZ70 is described as BSPH1, when in fact, it is a BSPH2 (Figs 1 and 5). Moreover, this ferret BSPH2 does not fulfill in the third group of the gene neighborhood pattern described by some BSPH2s (Fig 5, BSPH2 box), in which BSPH2/BSPH1 are not related with ELSPBP1 and a new gene appears (PLA2G4C, PhosphoLipase A2 Group IVC) not found in the above-described patterns. This pattern is present in mouse, rabbit, Tasmanian devil and rat. In the latter, new discrepancies between NCBI and Genomicus were found (Fig 5, BSPH2 box, 1.Rn.G and 1.Rn.N, respectively), focused on the absence of BSPH1 (UniProt code: B7ZWD1) in Genomicus. In addition, Genomicus ascribed G3VIB3 in Tasmanian devil GL850016.1 scaffold to a BSPH1 (Fig 5, BSPH2 box, GL8500161.Sh.G), when in fact it is a BSPH2. These new and misannotated sequences are also compiled in Table 1.

### Expression of RSVP20 and binding of the recombinant protein to spermatozoa

In order to validate the above *in silico* studies, the gene corresponding to *Ovis aries* RSVP20 was cloned and expressed in *E*. *coli*, since no previous BSP5 protein has been cloned, probably given their tendency to aggregate as inclusion bodies (IB). Using yeast SUMO (a small ubiquitin-like modifier) as a tag, the RSVP20 expression degree was high. Following IB solubilisation, RSVP20 was refolded on column and purified by affinity chromatography. SDS-PAGE analysis (S3 Fig) revealed the presence of RSVP20 in the flow-through (FT), where a high urea concentration occurs, and in two elution fractions. In the first elution fraction (E1), which corresponds to elution with 100 mM imidazole, RSVP20 was observed with other smaller bands. However, these bands were not present in the E2 fraction (elution with 250 mM imidazole), giving rise to purer fraction.

**Fig 6:**
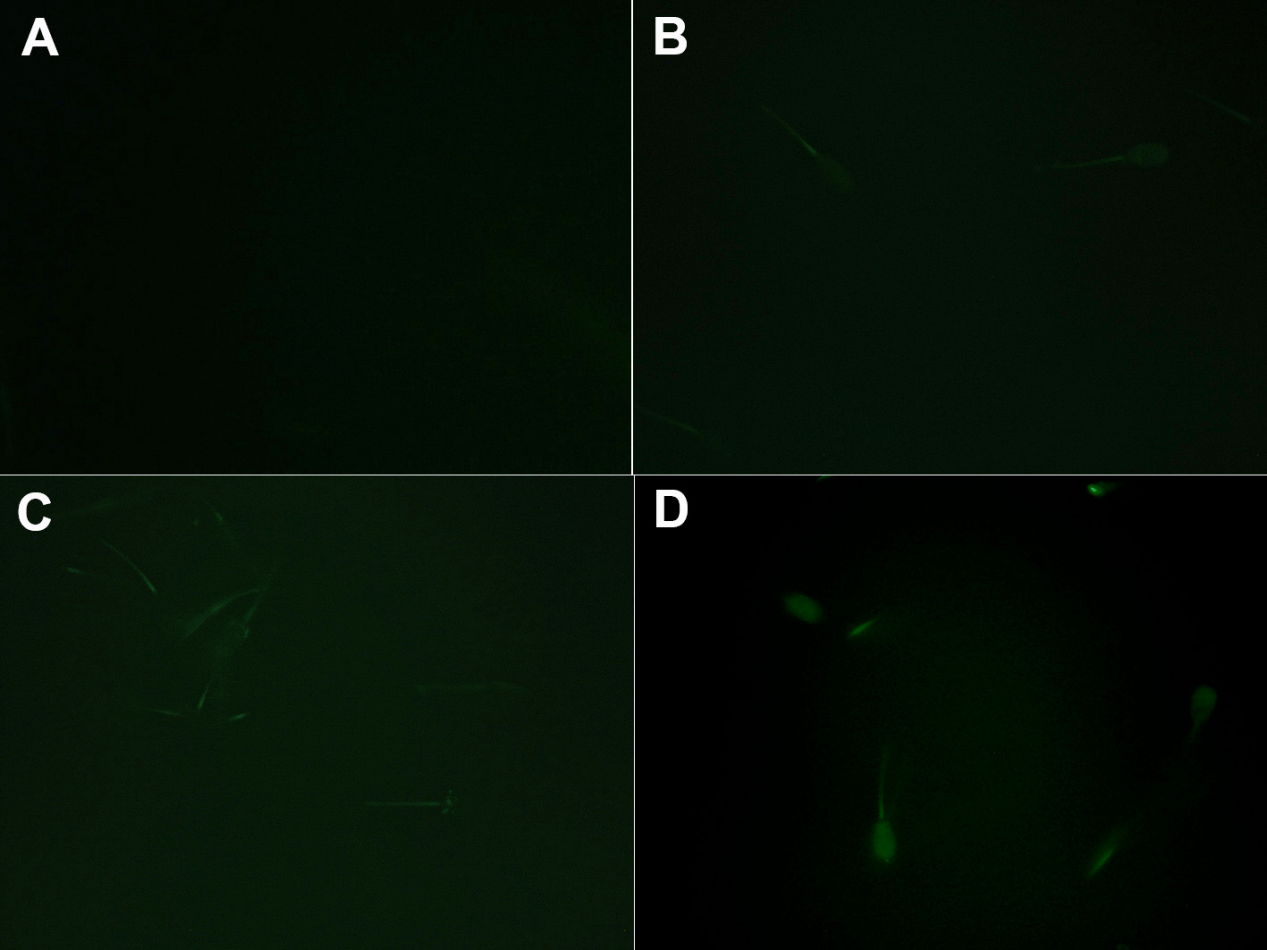
Fluorescence microscopy localization of the purified recombinant RSVP20 conjugated with Alexa Fluor 488 in fresh ram spermatozoa. A) Negative control of sperm samples incubated with 100 μL of the eluate obtained after passing Alexa Fluor 488 diluted with PBS through a size-exclusion spin column and filter. Concentration of Alexa-conjugated RSVP20 per 4 x 10^7^ cells: B) 3 μg; C) 7.5 μg and D)15 μg. Epifluorescence illumination using a B-2A filter at 400x magnification.

**Fig 7:**
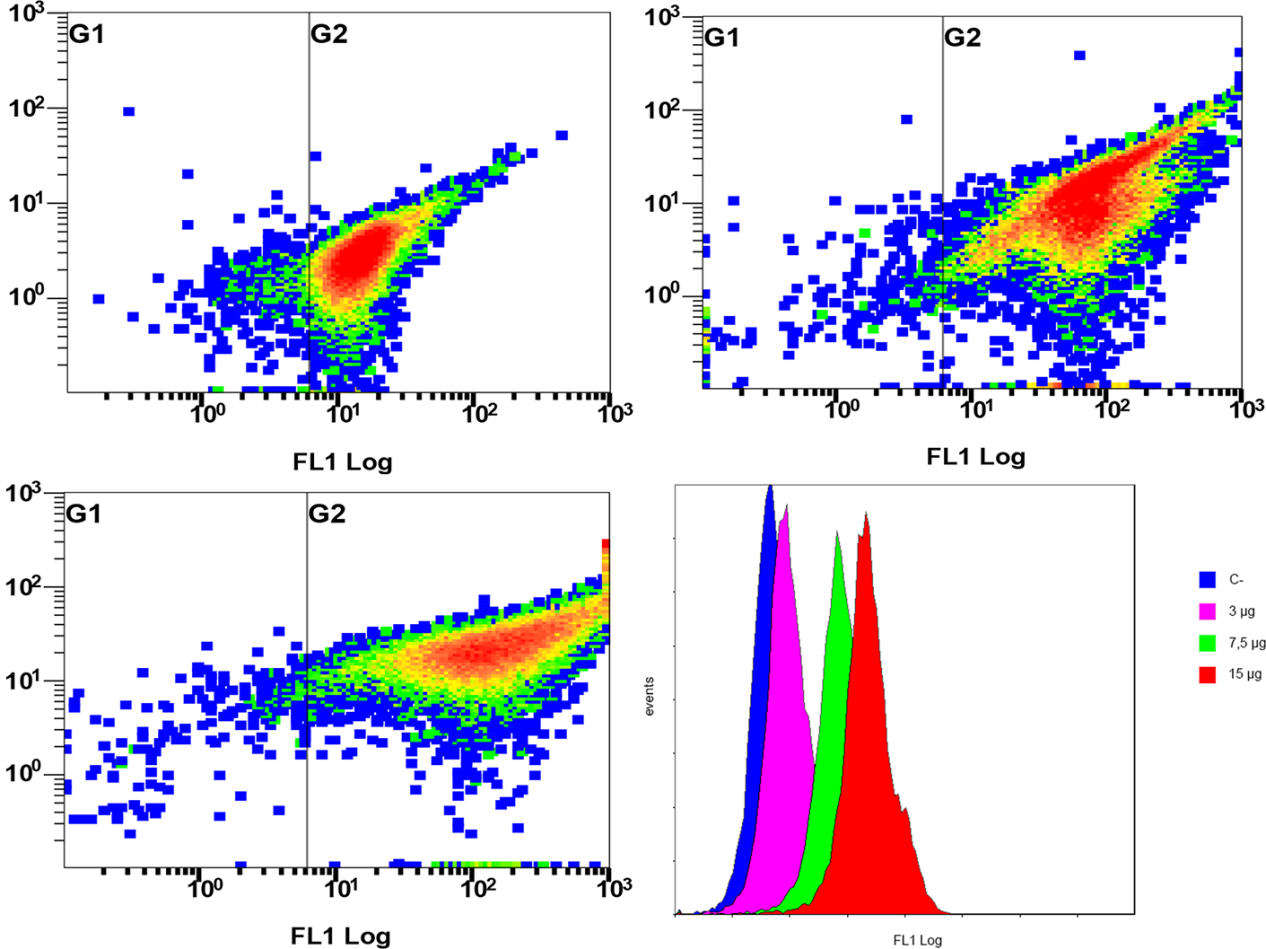
Flow cytometry analysis of the sperm binding capacity of Alexa Fluor 488-conjugated RSVP20. Cytometry analysis of the effect of sperm incubation with increasing concentration of Alexa-conjugated RSVP20: A) 3 μg; B) 7.5 μg; C) 15 μg per 4 x 10^7^ cells. D) Plots showing the number of cells linked with protein (events) and the different fluorescence intensity (FL1 Log) for increasing concentrations of Alexa-conjugated RSVP20.

**Fig 8:**
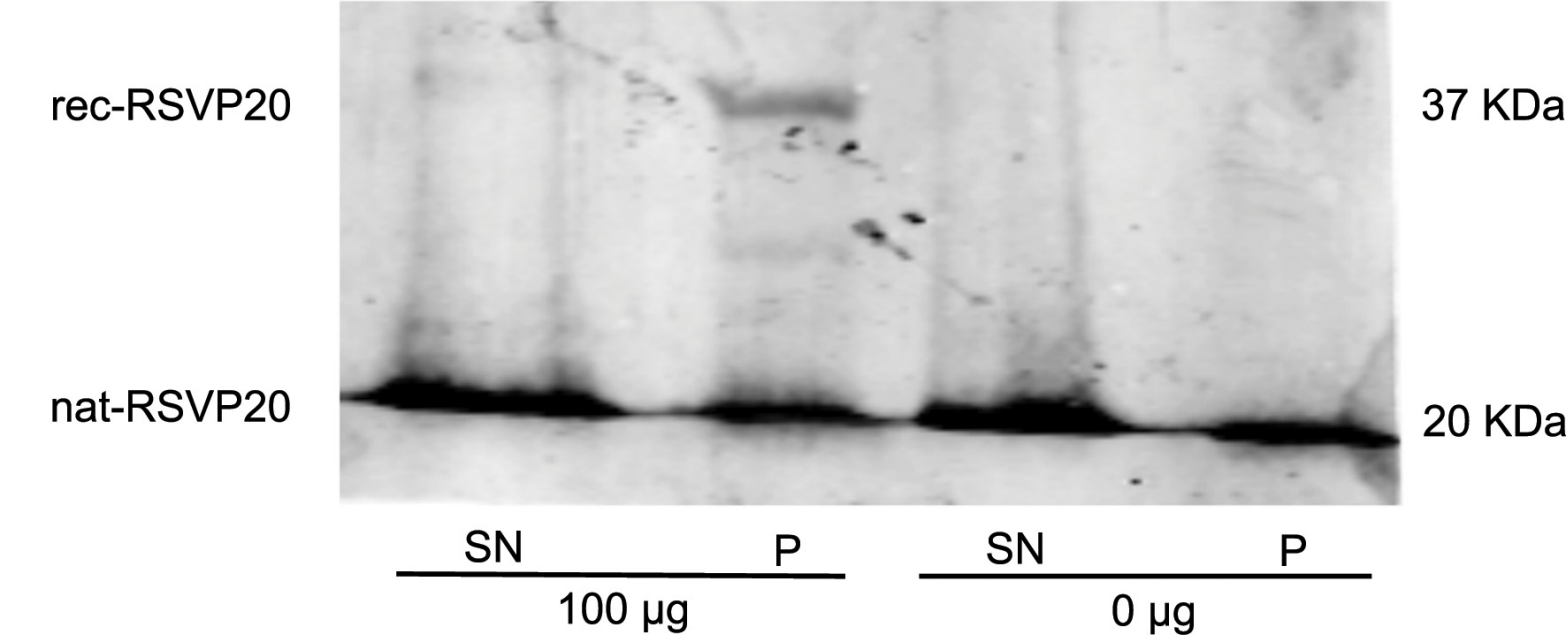
Western blot membrane indicating the presence of recombinant and native RSVP20 in ram sperm lysates. Supernatant fraction (SN) and pellet fraction (P) of sperm samples incubated with (100 ug) or without (0 ug) recombinant RSVP20.

The sperm binding capacity of the above-purified recombinant RSVP20 was tested incubating ram spermatozoa freed from seminal plasma by a dextran/swim-up procedure with increasing concentrations of Alexa-conjugated RSVP20. Fluorescence microscopy analysis evidenced the presence of RSVP20 on the sperm surface in a concentration-dependent manner (Fig 6). With the lowest protein concentration the flagella were preferably labelled (Fig 6B), and head staining was increasing with higher concentrations (Fig 6C and 6D). Control sperm samples incubated with the fluorophore diluted with PBS and passed through a size-exclusion spin column and filter resulted in a total absence of reactivity (S4 Fig and Fig 6A). These results were proved by flow cytometry analysis, which showed that RSVP20 binds to spermatozoa with a concentration-dependent effect. Higher protein concentration caused an increase in the quantity of adsorbed protein in the whole sperm population (Fig 7). In order to prove that the labelling was not due to the presence of potential traces of the non-conjugated dye (free fluorophore), sperm samples were incubated with 25, 50 or 100 μL of the eluate obtained after passing Alexa Fluor 488 diluted with PBS through a size-exclusion spin column and filter. No signal was detected at all by both fluorescence microscopy and flow cytometry analyses (see S4 Fig). In addition, the presence of RSVP20 in the sperm membrane was also confirmed by Western blotting of sperm lysates (Fig 8) that revealed a band of approximately 37 kDa, the corresponding molecular weight for this protein.

## Discussion

The phylogenetic analysis described in the present report (Fig 1 and S1 Fig) shows an up-dated picture of known BSPs proteins (BSP, BSPH1 and BSPH2) in protein databases (UniProt, NCBI and Ensembl). Sixty-four sequences have been used compared with the eighteen previously published [13,14]. The new sequences revealed a different evolutionary scenario of BSP families. Previous works showed two distinct clades, the BSP and the BSPH1/BSPH2, respectively [13,14]. However, the updated results (Fig 1 and S1 Fig) show the same three families but with different associations, i.e., BSPH1/BSP and BSPH2. This new tree topology is in agreement with the evolution scenario proposed for the BSP family by Tian et al (2009) [19], in which an early duplication event of a BSP family ancestor gave rise to a BSPH1/BSPH2 ancestor before the divergence of mammals, while a further independent duplication in ungulates from BSPH1 resulted in a BSP ancestor [19]. This plausible scenario is supported by the conserved block analysis (Fig 2) in which BSPH1 seems to be the most conserved pattern with three clear fingerprints (Block III, Block VI and Block VIII, respectively), whereas only one was detected in BSPH2 (Block IX) and none in BSPs. The absence of fingerprints in the latter indicates a high evolutionary pressure in BSP proteins to fulfil a new specific function during capacitation, different from those already carried out by BSPH1/BSPH2. This independent evolution could be favoured by the different chromosome location of BSP proteins as shown in the gene context analysis (Fig 5). In addition, the latter analysis showed three clear gene context patterns in the BSP family. The first is associated with BSPs and is closely related to the CD177 and TEX101 genes. In the second, BSPH1/BSPH2 coexists with the ELSPBP1 gene. In the third, BSPH1/BSPH2 is not related with ELSPBP1 but with PLA2AG4C, a calcium-independent Group IVC phospholipase A2. The biological role of these genes in reproduction has yet to be investigated. In addition, the combination of the detailed study of conserved blocks (Fig 2) to determine clear fingerprints for BSP, BSPH1 and BSPH2, together with the gene context analysis (Fig 5) could permit a correct assignment of new incoming BSP family sequences and the update of the high number of missanotated sequences and discrepancies in gene context found in current databases.

Cloning and expression of the RSVP20 gene validated these *in silico* studies, this being the first time that a BSP5 protein has been cloned. Binding assays proved that the purified recombinant protein maintains the ability to get adsorbed onto the surface of all the spermatozoa in the sample, independently of possible differences in their membrane [20–22]. The tendency of these proteins to aggregate as inclusion bodies has tried to be avoided for other BSP family proteins by using both a thioredoxin N-ter tag (i.e., pET32a expression vector) and an *E*. *coli* stain engineered for producing recombinant proteins with disulfide bonds (i.e., Rosetta-gami B) [7], but the main method of purifcation is by IB solubilization [8,23]. In this study, the use of yeast SUMO resulted in a high degree of expression of RSVP20, independently of the temperature and IPTG concentration. The protein was accumulated in the insoluble fraction forming inclusion bodies (IB) from which RSVP20 was purified following an IB solubilizing approach that allowed the extraction of 80% of the proteins after 30 min incubation and a on column protein refolding strategy. Therefore, our results suggest that the methodology used in this study can be useful for cloning and expression of other BSP proteins.

In conclusion, this study gives up a new picture of BSP family describing and correctly assigning sequences from known databases (UniProt, Ensembl and NCBI), which in combination with known cloning technology or using synthetic gene synthesis could permit a further biochemical and functional characterization of the BSP protein family in sperm capacitation and functionality, as we have been shown with RSVP20. Obtaining purified recombinant BSPs will aid the understanding of the biological role of the BSP protein family.

## Materials and Methods

### Phylogenetic and *in silico* analysis

BSP sequence homologies were identified using the NCBI BLAST algorithm using RSVP20 as entry. Each sequence found was checked again by BLAST in order to find new members in UniProt. ELSPBP1, MMP9, incomplete sequences and duplicates were removed, rendering the sequences described in Table 1. A neighbour-joining (NJ) tree analysis was performed using the MEGA6 software with pairwise deletion options and the Dayhoff PAM matrix model. A bootstrap support value for NJ tree was obtained from 1000 replicates. The Interactive Tree of Life (iTOL) was used for the display and manipulation of the phylogenetic trees [24]. Protein sequences were 3D modelled with Geno3D [25]. Docking was performed with SwissDock [26] and molecular visualization was performed with PyMOL [27]. Conserved blocks were detected using WebLogo3 [16] and ESPript [17].

### Amplification and sequencing of cDNA encoding RSVP20

This study was carried out in strict accordance with the Guide for the Care and Use of Laboratory Animals of the European Union Directive. The Committee on the Ethics of Animal Experiments of the University of Zaragoza approved the protocol.

Tissue samples from seminal vesicles were collected from a freshly slaughtered *Rasa Aragonesa* male ram (*Ovis aries*) and immediately frozen in liquid nitrogen. Total RNA was extracted by the guanidine thiocyanate/phenol extraction method [28,29] by homogenization in 1 mL of TRI reagent (Sigma-Aldrich) per 200 mg of tissue. RNA concentration was measured in a NanoDrop ND-100 Spectrophotometer (Wilmington, DE, USA). 500 ng of total RNA was reverse transcribed using poly (dT) primers and the SuperScript III RT enzyme (Invitrogen, CA, USA).

Degenerate PCR primers (S1 Table) for RSVP20 were designed according to the primary sequence of the protein [30] in the 5’ region. Primer RSVP20-5’ was based on amino acids residues 1–6. The 3’ primer was Oligo (dT)_20_. PCR was performed with 2 μL of cDNA. Using these primers, PCR (35 cycles) were carried out on reverse-transcribed RNA from ram seminal vesicles. Cycling conditions consisted of 45 s at 94°C, 1 min and 30 s at 53°C, and 3 min at 72°C. A 1 min denaturation step at 94°C preceded cycling; at the end, a final 10 min extension at 72°C was performed. PCR products were separated on 2% agarose gel in 1x Tris-borate-EDTA (TBE) buffer containing 0.5 μL/mL ethidium bromide and were visualized under ultraviolet (UV) light. Molecular size was estimated by using GeneRuler 1kb plus (Thermo Scientific). PCR products were gel-purified using a GeneJet gel extraction kit (Thermo Scientific), in accordance with the manufacturer’s instructions and sequenced on an ABI Prism 3730 sequencer (Applied Biosystems, Foster City, CA, USA).

### Cloning of cDNA sequences into *E*. *coli*

RSVP20 was cloned into pE-SUMO3 plasmid, which contained both a His-tag and a SUMO-tag, using primers 20*BbsI*-Fw and 20*XbaI*-Rv (S1 Table). The plasmid pE-SUMO3-RSVP20 was transformed into Origami B (DE3)pLysS competent cells. Transformed bacteria were grown overnight on LB-agar plates containing 50 mg/L ampicillin and 15 mg/L kanamycin. Selected colonies were grown in 5 mL LB media with 50 mg/L ampicillin and 15 mg/L kanamycin to an OD_600_ of 0.6–0.8. Protein expression was induced by adding isopropyl-β-D-thiogalactoside (IPTG) to final concentrations of 0.5 and 1 mM. Inductions were performed at 37°C for 8 h and 20°C for 16 h. One mL culture samples were collected before and after the IPTG induction. *E*. *coli* cells were harvested from the samples by centrifugation at 6800 x g for 2 min at 4°C, resuspended in lysis buffer (50 mM Tris-HCl, 300 mM NaCl, 10% B-PER, lysozyme 1mg/mL, pH 8.0) and lysed by sonication. The lysate was centrifuged for 5 min at 6500 x g and the resulting pellet was resuspended in lysis buffer. Both the pellet and the supernatant were checked for expression.

### Nickel affinity chromatography and refolding of RSVP20

Purification of RSVP20 was carried out from inclusion bodies (IB) using nickel affinity chromatography. *E*. *coli* cells were grown in 1 L of TB (Terrific broth) medium to an OD_600_ of 4.0. Protein expression was induced with 0.5 mM of IPTG for 16 h at 20°C. Afterwards, the cells were harvested by centrifugation at 6500 x g for 15 min at 4°C and the cell pellets were resuspended in lysis buffer and lysed by sonication. The lysate was centrifuged for 15 min at 6500 x g and the pellet was washed twice, first with lysis buffer and then with sample buffer (20 mM Tris, 500 mM NaCl, 5 mM imidazole pH 7.5) containing 2 M urea. The final pellet was resuspended in denaturing buffer (sample buffer with 8 M urea and 10 mM β-mercaptoethanol). Soluble and insoluble fractions were separated by centrifugation at 40000 x g for 40 minutes and the soluble fraction was loaded into a His-TrapFF column (GE Healthcare) equilibrated with denaturing buffer at a flow rate of 1 mL/min. The column was then washed with 5 bed volumes of denaturing buffer and 5 volumes of washing buffer (20 mM Tris, 500 mM NaCl, 80 mM imidazole, 8 M urea, 10 mM β-mercaptoethanol, pH 7.5). Refolding of the bound protein was performed with an on-column decreasing linear gradient of 7 mM urea/min from denaturing buffer to sample buffer. The refolded proteins were eluted successively with elution buffer containing two different imidazole concentrations (20 mM Tris, 500 mM NaCl pH 7.5, with 100 mM and 250 mM imidazole, respectively).

### Fluorescent labeling and binding of RSVP20 to spermatozoa

Recombinant RSVP20 was conjugated with Alexa Fluor using Alexa Fluor 488 Microscale Protein Labeling Kit (Life Technologies) following the manufacturer’s instructions. Briefly, 100 μg of protein (1 μg/μL) were incubated with the dye for 15 minutes. Afterwards, non-conjugated dye was removed using the size-exclusion spin columns (Bio-Gel P-6) and the filters provided with the kit.

Final protein concentration was calculated using the formula:

$$\text{Protein concentration}\;\left(\text{M}\right)=\frac{\left[{\text{A}}_{280}-\left({\text{A}}_{494}\;\times \;0.11\right)\right]\times \text{dilution factor}}{45,380}$$

where 45,380 is the theoretical molar extinction coefficient (ε) in cm^–1^ M^–1^ of RSVP20 at 280 nm and 0.11 is a correction factor for the fluorophore’s contribution to the absorbance at 280 nm. The obtained protein concentration was 0.15 mg/mL.

Fresh ram semen was collected from 4 mature *Rasa aragonesa* rams using an artificial vagina. The rams belonged to the National Association of Rasa Aragonesa Breeding (ANGRA) and were 2 to 4 years old. They were kept at the Experimental Farm of the University of Zaragoza (Spain) under uniform nutritional conditions in compliance with the requirements of the European Union Directive for Scientific Procedures. A seminal plasma–free sperm population was obtained by a dextran/swim-up procedure (García-López et al, 1996) performed at 37°C using a medium without Ca_2_Cl and NaHCO_3_ (Pérez-Pé et al, 2002). Sperm concentration was calculated in duplicate with a Neubauer’s chamber (Marienfeld, Germany). 4 x 10^7^ cells in a final volume of 500 μL completed with PBS were used for all the experiments.

The binding of different concentrations of Alexa conjugated RSVP20 (3 μg, 7.5 μg and 15 μg) was detected by fluorescence microscopy using a Nikon Eclipse E400 microscope (Nikon, Tokyo, Japan) with a B-2A filter (excitation 450–490 nm) at 400x magnification, and flow cytometry using an equipment (Beckman Coulter FC 500, IZASA, Barcelona) with a CXP software, equipped with two lasers of excitation (Argon ion laser 488 nm and solid state laser 633 nm) and five filters of absorbance (FL1-525, FL2-575, FL3-610, FL4-675, and FL5-755; 65 nm each band pass filter). 4 x 10^7^ spermatozoa were incubated at room temperature (RT) in darkness for 15 min with different concentrations of RSVP20 (final volume 500 μL), then washed with 500 μL PBS at 600 xg for 5 min. The final pellet was resuspended in 500 μL PBS and samples were mounted onto microscope slides or analyzed by flow cytometry. At a minimum, 20,000 events were counted in all cytometry experiments. The sperm population was gated for further analysis on the basis of its specific forward (FS) and side scatter (SS) properties; other non-sperm events were excluded. A flow rate stabilized at 200–300 cells per second was used. Monitored parameters were FS log, SS log, and FL1 (Alexa Fluor 488).

In order to prove that the labelling was not due to the presence of potential traces of the non-conjugated dye (free fluorophore), the content of another fluorophore vial was diluted with 100 μL PBS instead of the protein sample, and passed through a new size-exclusion spin column and filter provided with the kit. Sperm samples were incubated with the same volumes of this eluate as those used in the binding protein assays (20, 50 and 100 μL), and fluorescence microscopy and flow cytometry analyses were carried out.

To study the binding of RSVP20 to sperm membranes by Western blot, 4 x 10^7^ cells freed from seminal plasma were incubated 15 min at RT in the presence or absence of 100 μg of RSVP20 in a final volume of 500 μL. Following incubation, the unbound protein was removed by centrifuging at 600 xg for 8 min. Then, the supernatant was removed and the pellet was washed with 300 μL of PBS and centrifuged again. The pellet was resuspended with 100 μL of PBS and 100 μL of extraction buffer (125 mM Tris-HCl, 4% SDS) and, after incubation at 100°C in a sand bath for 5 min, it was centrifuged again at 12,000 xg for 5 min. A mix of 10% of a protease and phosphatase inhibitor cocktail (Sigma-Aldrich), 10% β-mercaptoethanol, 20% glycerol, and 0.02% bromophenol blue were added to all recovered fractions, and the samples were analyzed by SDS-PAGE and Western blot.

SDS-PAGE was performed in 14% polyacrylamide gel using a Mini protean III system (Bio-Rad, Hercules, CA, USA). Electrophoresis was performed for 90 min at 130 V at 4°C. A mixture of pre-stained protein standards (Bio-Rad, Hercules, CA, USA) was used as a marker. The proteins were transferred to a polyvinylidene difluoride (PVDF) membrane using the Trans-blot Turbo (Bio-Rad, Hercules, CA, USA). The transference was performed for 10 min at 2.5 A-25 V and the membrane was air dried for 15 min. Non-specific sites on the membrane were blocked for 1 h with 5% BSA in PBS. RSVP20 was detected by incubating overnight at 4°C with specific rabbit generated antibodies [31] diluted 1:60,000 in PBS with 1% BSA and 1% Tween. After exhaustive washing, the membranes were incubated with a secondary anti-rabbit Dylight 680 conjugated (Thermo Scientific, Madrid, Spain) diluted 1:15,000 for 1 h at RT. After washing, the membrane was scanned using Odyssey Clx (Li-Cor Biosciences, Lincoln, NE, USA).

## Supporting Information

S1 Fig NJ phylogenetic tree of Binder of Sperm Proteins. The numbers indicate the NJ bootstrap values for 1000 replicates (see Material and Method for details). Only bootstrap values larger than 50% are shown and bold numbers indicate the three main families. UniProt and NCBI codes are listed in Table 1.
(TIF)

S2 Fig Amino acid sequence of RSVP20. ESPript output obtained with the modeled RVSP20 protein retrieved from Uniprot database and later aligned with murine BSPH1 and murine BSPH2 using CLUSTAL-W. Residues strictly conserved are in red. Symbols above blocks of sequences represent the secondary structure, springs represent helices and arrows represent β-strands. The signal peptide is in a box. The disulfide bonds in each FN2 domain are indicated with italic numbers, 1–2 for 1FN2 and 3–4 for 2FN2 domains.
(TIF)

S3 Fig SDS-PAGE analysis of recombinant RSVP20 purification and on-column refolding process. SN, supernatant after incubation with 8 M urea and 10 mM β-mercaptoethanol; FT, flow through onto a nickel affinity chromatography; W, wash; E1, elution with 100 mM imidazole; E2, elution with 100 mM imidazole.
(TIF)

S4 Fig Fluorescence microscopy localization (A, B, C, D) and Flow cytometry analysis (E, F, G, H) of sperm samples incubated with 0 μL (A, E), 25 μL (B, F), 50 μL (C, G) or 100 μL (D, H) of the eluate obtained after passing Alexa Fluor 488 diluted with PBS through a size-exclusion spin column and filter. Epifluorescence illumination using a B-2A filter at 400x magnification. Flow cytometry plots showing the number of cells (events) and the different fluorescence intensity (FL1 Log) for increasing concentrations of Alexa-conjugated RSVP20.
(TIF)

S1 Table Oligonucleotide sequences used for PCR amplifications and sequencing. (DOCX)
