# Supplementary figures and images for: New Insights into the Phylogeny and Gene Context Analysis of Binder of Sperm Proteins (BSPs)
Source: PLoS One. 2015 Sep 2;10(9):e0137008. doi: 10.1371/journal.pone.0137008 (PMC4557993; doi:10.1371/journal.pone.0137008)

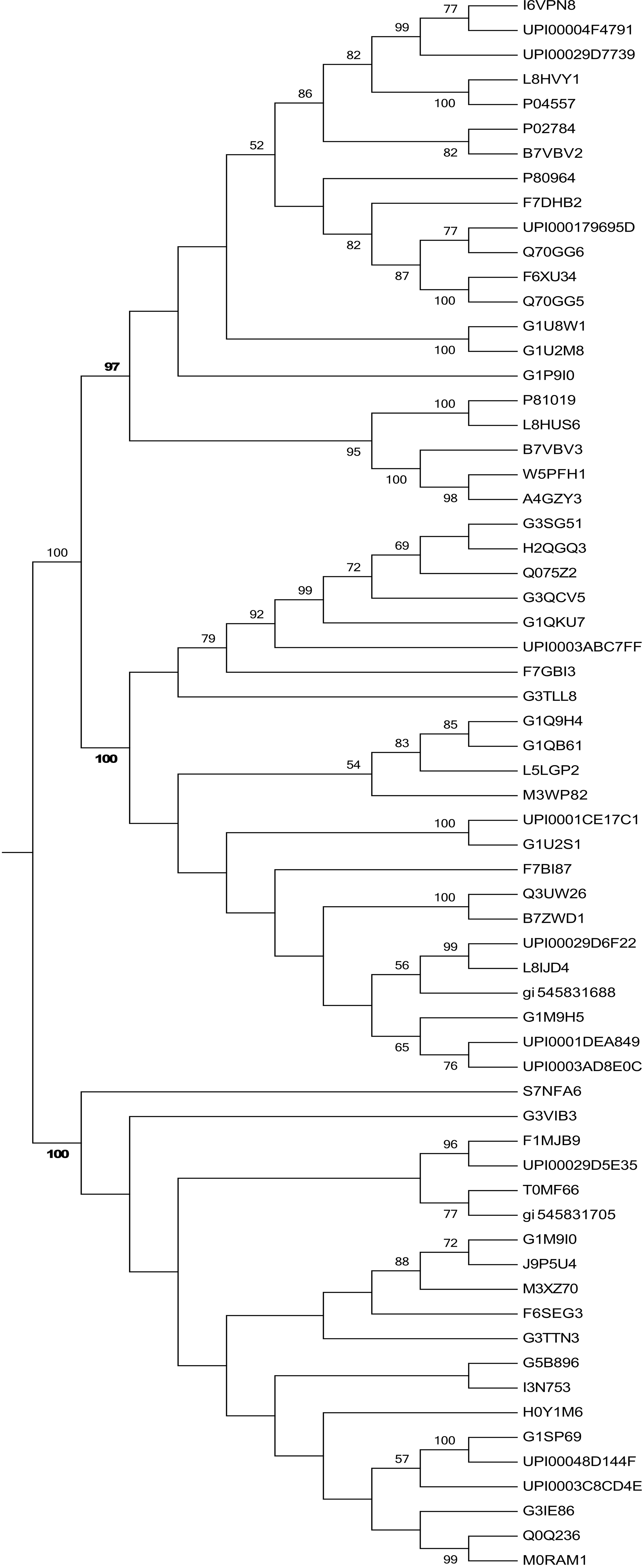

Supplement: S1 Fig — The numbers indicate the NJ bootstrap values for 1000 replicates (see Material and Method for details). Only bootstrap values larger than 50% are shown and bold numbers indicate the three main families. UniProt and NCBI codes are listed in Table 1. (TIF) [file pone.0137008.s001.tif]

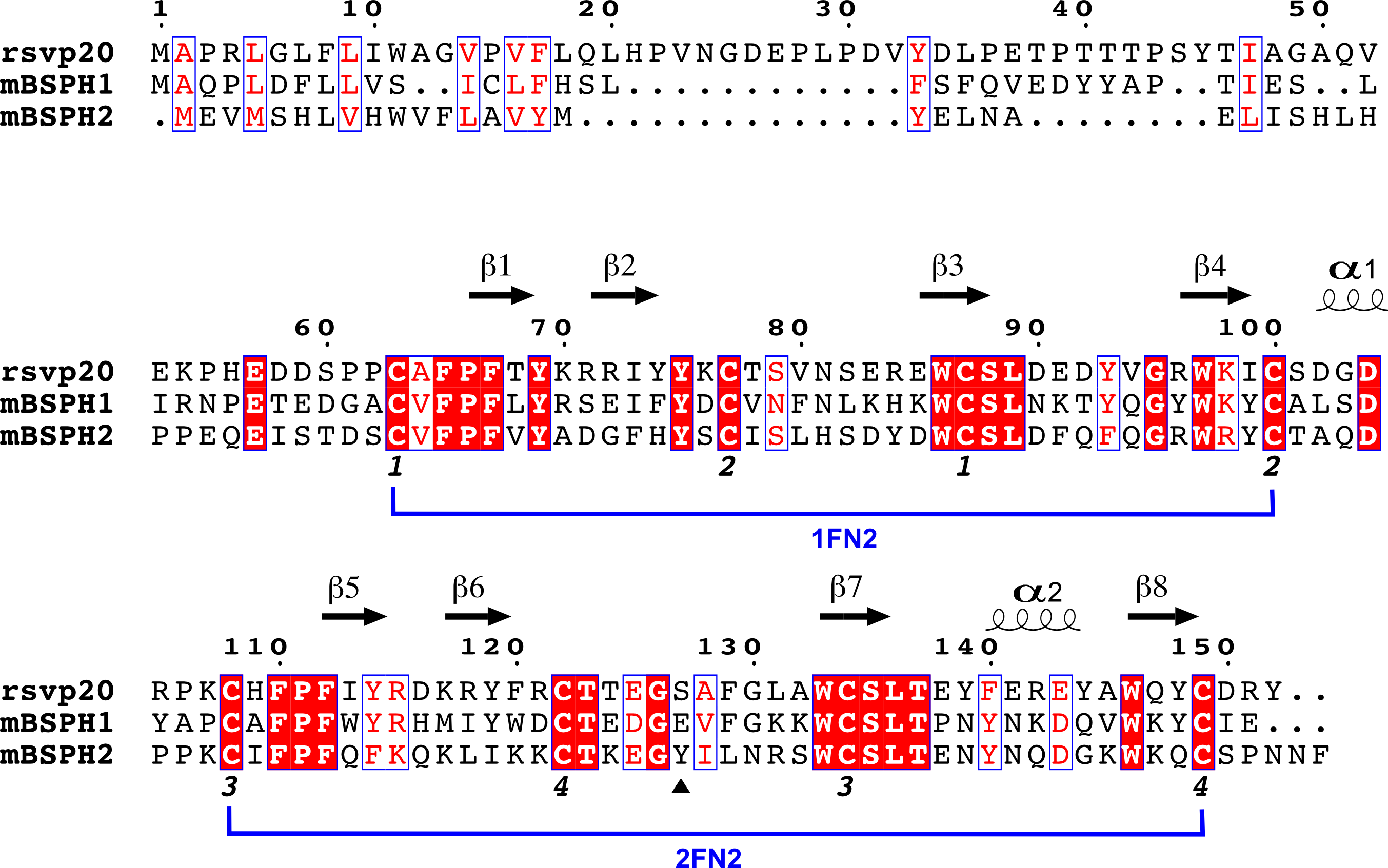

Supplement: S2 Fig — ESPript output obtained with the modeled RVSP20 protein retrieved from Uniprot database and later aligned with murine BSPH1 and murine BSPH2 using CLUSTAL-W. Residues strictly conserved are in red. Symbols above blocks of sequences represent the secondary structure, springs represent helices and arrows represent β-strands. The signal peptide is in a box. The disulfide bonds in each FN2 domain are indicated with italic numbers, 1–2 for 1FN2 and 3–4 for 2FN2 domains. (TIF) [file pone.0137008.s002.tif]

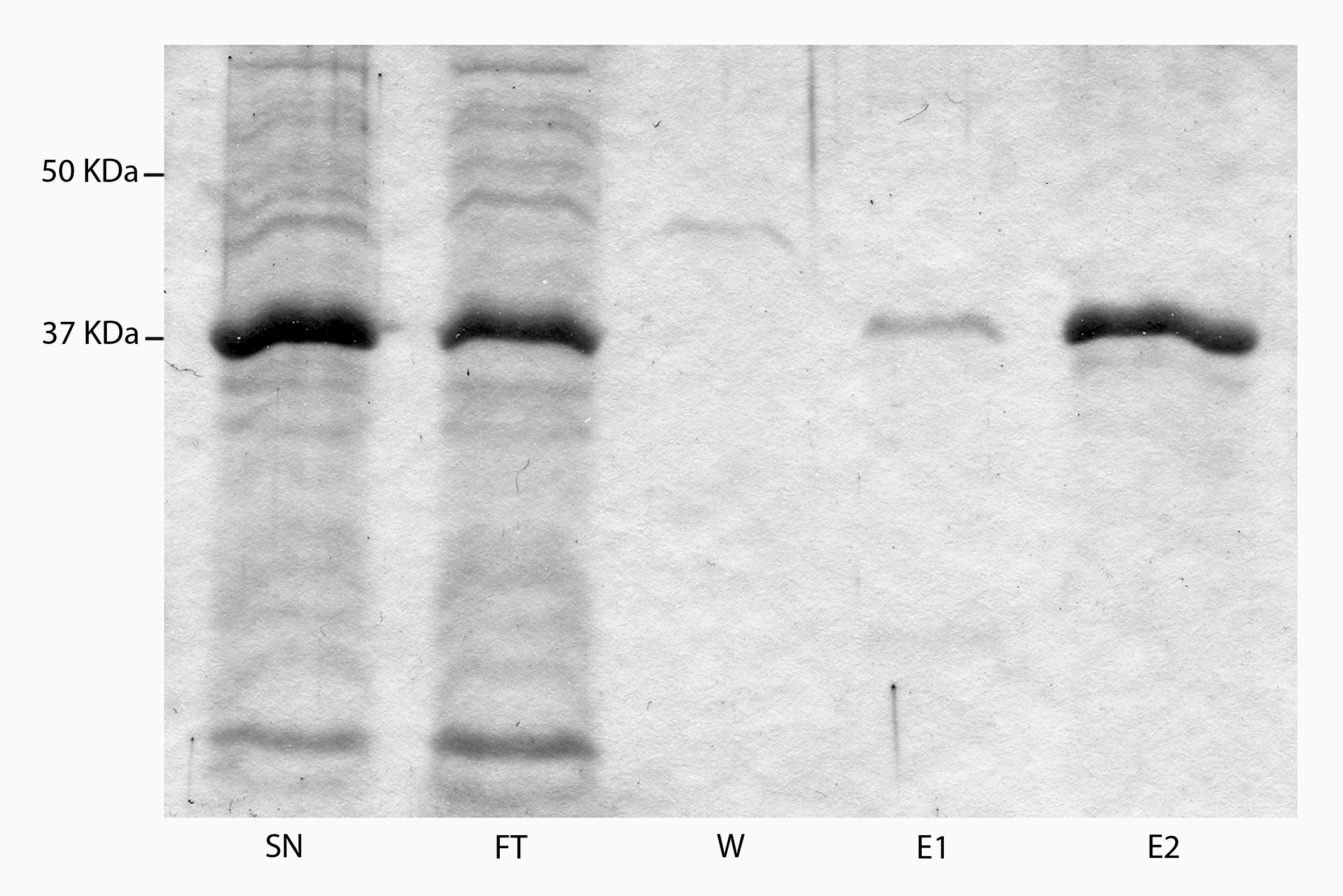

Supplement: S3 Fig — SN, supernatant after incubation with 8 M urea and 10 mM β-mercaptoethanol; FT, flow through onto a nickel affinity chromatography; W, wash; E1, elution with 100 mM imidazole; E2, elution with 100 mM imidazole. (TIF) [file pone.0137008.s003.tif]

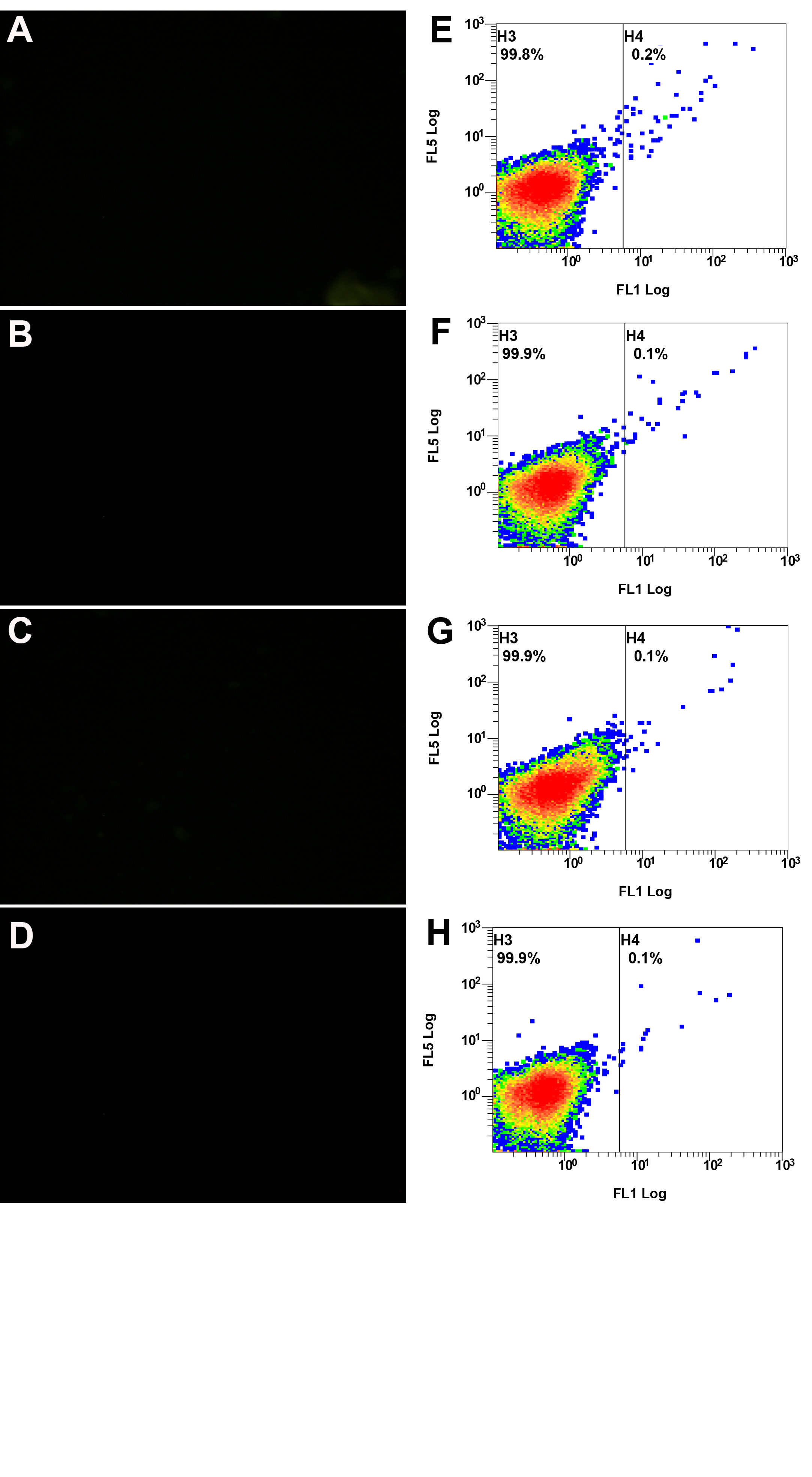

Supplement: S4 Fig — Epifluorescence illumination using a B-2A filter at 400x magnification. Flow cytometry plots showing the number of cells (events) and the different fluorescence intensity (FL1 Log) for increasing concentrations of Alexa-conjugated RSVP20. (TIF) [file pone.0137008.s004.tif]
