# Supplementary material for: New Insights into the Phylogeny and Gene Context Analysis of Binder of Sperm Proteins (BSPs)
Source: PLoS One. 2015 Sep 2;10(9):e0137008. doi: 10.1371/journal.pone.0137008 (PMC4557993; doi:10.1371/journal.pone.0137008)
Supplement: S1 Table — (DOCX) [file pone.0137008.s005.docx]

| **S1 Table. Oligonucleotide sequences used for PCR amplifications and sequencing*.** Sequences recognized by corresponding restriction enzymes (in cursive in the name of the primers) were included*.* | |
| --- | --- |
| RSVP20-5’ | 5’-GAT GAI CCI CTI CCI GA-3’ |
| 20comp-Fw | 5’-ACA GGA GAG CAA GCT ACC A-3’ |
| 20comp-Rv | 5’-ATT GAC TGG ACC CAT GCA CAG -3’ |
| 20*Sgfl*-Fw | 5’-ATC GGC GAT CGC CAT GGC ACC GCG TCT GGG GCT -3’ |
| 20*Pmel*-Rv | 5’-ATC GGT TTA AAC TTA ATA CCG ATC GCA GTA CT -3’ |
| 20*BbsI*-Fw | 5’-AAG AAG ACT AAG GTA TGG CAC CGC GTC TG-3’ |
| 20*XbaI*-Rv | 5’-GGT CTA GAT TAA TAC CGA TCG CAG TA -3’ |
| *Oligonucleotide primers were synthesized at Invitrogen Inc. I indicates inosine. | |
